# Dial-a-molecule workshop: computational prediction of reaction outcomes and optimum synthetic routes

**DOI:** 10.1186/s13065-015-0129-9

**Published:** 2015-09-23

**Authors:** Kelly J. Kilpin, Jonathan M. Goodman, A. Peter Johnson, Richard J. Whitby

**Affiliations:** Department of Chemistry, University of Southampton, Southampton, SO17 1BJ UK; Department of Chemistry, Centre for Molecular Informatics, Lensfield Road, Cambridge, CB2 1EW UK; Department of Chemistry, University of Leeds, Leeds, LS2 9JT UK

**Keywords:** Computational, Reaction prediction, Optimisation, CASD

## Abstract

Computational prediction of reaction outcomes and optimum synthetic routes was a two-day meeting and workshop organised by the EPSRC Dial-a-molecule grand challenge network. Forty delegates discussed computer predictions of synthetic routes and reactions, and considered their relevance to contemporary chemistry.

**Graphical abstract:**
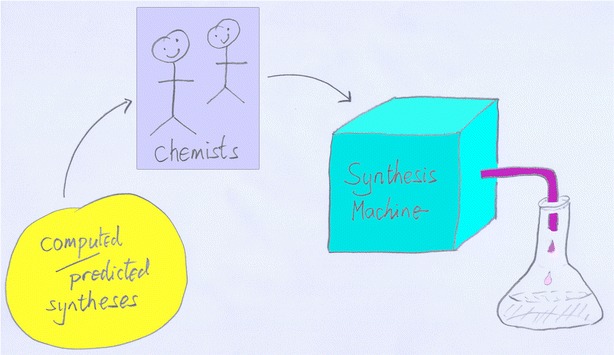
Dial-a-molecule workshop: computational prediction of reaction outcomes and optimum synthetic routes.

Dial-a-molecule workshop: computational prediction of reaction outcomes and optimum synthetic routes.

## Background

We are now living and working in an information age—but is synthetic chemistry being left behind? Are we taking full advantage of the large amounts of experimental data now being routinely generated and the massive computing power now available to enrich and manipulate it? This question brought forty members of the Dial-a-Molecule community together in Leeds, United Kingdom in September 2014.

The meeting was opened by Richard Whitby (University of Southampton, Dial-a-Molecule PI) who set the scene for the following two days, by describing the vision of the Dial-a-Molecule Grand Challenge: “In 20–40 years, scientists will be able to deliver any desired molecule, within a timeframe useful to the end-user, using safe, economically viable and sustainable processes.” Because synthesis is such a complicated, multi-faceted process ‘what is made’ is often limited by ‘what can easily be made’ using well established, trusted reactions where the outcome can be predicted with confidence before entering the lab. As a result, many of the molecules made on a day-to-day basis represent a compromise controlled by synthetic accessibility, and are not necessarily the best candidates for the job. A key step towards achieving the Grand Challenge, where making any molecule will be as simple as dialing a number, will be made when the chemist is able to reliably predict the outcome of unknown reactions. However, to achieve this goal, synthesis must move toward becoming a data-driven discipline that takes full advantage of the on-going computing and automation revolution.

The two-day meeting consisted of presentations detailing the methods chemists currently use to predict reaction outcomes and the tools available to design synthetic routes. Breakout sessions were structured around three key discussion points: predicting reaction outcomes, designing synthetic routes and driving the development of new chemistry.

## Session 1: predicting reaction outcomes

The meeting began with a series of talks on analysing individual reactions. A thorough understanding of the mechanistic pathway of a reaction can help ensure that reactions work as expected. Many techniques have been used to delineate reaction mechanisms, and computational approaches are particularly useful. Ian Fairlamb (University of York) showed that the mechanism of palladium coupling reactions is much more complicated than taught at an undergraduate level. Work from his group has led to improved understanding of the role that palladium nanoparticles play in the catalytic cycle of Stille cross-coupling reactions. John Slattery (University of York) described how DFT, combined with experimental evidence from in situ spectroscopic techniques, provides insight and a method for optimising complex catalytic processes. Andrei Malkov (Loughborough University) explained how quantum chemistry has played a critical role in unraveling the mechanism of catalytic asymmetric crotylation reactions. Natalie Fey (University of Bristol) showed how maps of the chemical space covered by phosphorus donor ligands in transition-metal catalysed reactions are extremely powerful, both to interpret data, and to predict reactivity.

Reaction optimisation need not be as time-consuming, expensive and repetitive as commonly perceived. Statistical tools, described by Dave Woods (University of Southampton) are underused by chemists, but, with the right knowledge, can optimise reactions efficiently. However, despite the increased use of computational and statistical tools to understand chemical transformations, Alexei Lapkin (University of Cambridge) pointed out that complex processes are still largely unpredictable, however well individual components are understood. For example, nanoparticles are increasingly used in a number of applications, but as Nguyen T K Thanh (University College London) discussed, reliable methods to routinely produce high-quality nanoparticles are scarce and molecular knowledge is required to predict their formation and to tune their size and morphology.

Many reactions and processes still need further investigation. The first breakout groups identified the most important are those that require scaling up and are sustainable (both atom economic and non-precious metal), whether commonly used or just emerging. Somewhat pleasing for synthetic chemists (particularly complacent ones), was the group consensus that no matter how much the computing power increases or technology develops, computer-aided synthesis design (CASD) or machines will never fully replace an experienced chemist. However, if the technology helps us to choose the right reaction conditions at the first attempt, a huge step towards the aims of Dial-a-Molecule will have been taken.

The groups concluded there is a large amount of confidence in predicting the outcomes of tried-and-tested reactions (e.g., amide/ester bond formation, CuCAAC ‘click’ reactions, hydrogenations, Pd-cross couplings), and the general conditions for a reaction to be trusted are those which have a broad and robust substrate scope, good functional group tolerance, and remain selective over a range of conditions and scales. In practice, many reactions do not meet these conditions and, too often, many of the reactions that are extracted from the databases or predicted via CASD do not proceed as expected—either the yields are too low for the reaction to be viable, time must be spent on optimisation procedures, or specific functional groups are incompatible with the reaction conditions.

## Session 2: designing synthetic routes

Even if individual reactions were perfectly understood, synthesis would remain a demanding task. When designing a synthetic route toward a particular target, a chemist will usually propose a (retro)synthetic pathway using their knowledge and experience. CASD is often used in this process, but human intervention and chemical knowledge are still required to verify the results. Peter Johnson (University of Leeds) gave a detailed history of CASD and described how reaction databases can be used to design synthetic routes. However, despite the advances in computing power and knowledge, the principles that were first laid down by Corey almost forty-five years ago are still relevant today. Indeed, modern tools such as ARChem (David Flanagan) and ICSynth (Mike Hutchings and Fernando Huerta) are based upon these rules, and can provide practical solutions to some everyday synthetic problems, and can also act as idea generators when planning synthetic routes. The field has progressed since Corey’s seminal paper [E J Corey and W T Wipke. *Science* 1969, 166, 178–192]; Anthony Cook (University of Leeds) described Computer-Aided *Enantioselective* Synthesis Design, and showed it is now possible to address stereochemical issues in automated synthesis planning. Mike Bodkin (Evotec) explained how reaction vectors are powerful tools in reaction analysis.

Once a CASD program generates a variety of synthetic routes, it must try to rank their quality. The requirements are subjective and so hard to automate. For example, some users may wish to select a route that limits the presence of impurities or products of side reactions. Nicole McSweeney (LHASA Ltd) described how LHASA is addressing automated risk assessment of mutagenic impurities, which has high relevance within the pharmaceutical industry. Regulatory authorities are averse to accepting risk assessments based solely on chemical arguments.

The second breakout groups concluded, once again, that despite being useful tools, CASD programs do not come without their problems and will never fully replace an experienced synthetic chemist. Problems associated with their use partly arises because the literature contains nearly exclusively positive results, and, therefore, the data used to feed CASD programs excludes much of the experience of skilled chemists. To overcome this drawback, these databases must be populated with high quality reaction data—Electronic Laboratory Notebooks (ELNs), where all data is recorded, are generally agreed to be the best source of this data, although the global academic adoption of ELNs is some way off.

When using CASD to generate multi-step retrosynthetic pathways, a combinatorial explosion is unavoidable and increases with the number of steps between the product and starting material. The impact of this can be minimised by focusing on a key transformation in the route or by pruning the output. However, like ranking routes, pruning criteria are subjective and chemical knowledge is required, alongside a computer interface to allow the user to define which factors are most important. By defining ranking and pruning criteria, the user creates a trade-off between practicality and creativity.

## Session 3: relevance to contemporary chemistry

Equipped with the tools to design ‘perfect’ synthetic routes and predict with 100 % confidence the outcome of reactions, how can we apply them to new chemistry? Is there still new chemistry to explore? Jonathan Goodman (University of Cambridge) described how we can already do a lot, and computation methods and InChI codes can help us order chemistry, but we can still do more with what we have. Mark Leach described how his ordering of chemical information is available on-line through his chemical thesaurus. Roger Sayle (NextMove Software) spoke about the breadth of information that is currently available in industrial ELNs, and gave examples of how reactions extracted from ELN’s can be categorised to give information on the most or least successful reactions.

The final breakout groups concluded that there is still much scope for developing new chemistry, particularly with respect to (re)designing reactions (e.g., using designer enzymes, tandem region/stereo selective reactions, avoiding protection and deprotection steps, converting stoichiometric reactions to catalytic) aimed towards synthesising useful families of targets molecules, such as 3D fragments or the top 200 drug molecules.

Armed with the ability to use ever expanding computing power to predict reaction outcomes and design better synthetic routes, the groups discussed the possibility of taking this technology and incorporating it into a machine capable of making compounds. Ideally, it would have to make a wide variety of different compounds and carry out diverse transformations—a step up from oligionucleotide synthesisers which just use three types of chemistry. In addition, to avoid gathering dust, it would have to be modular and include interchangeable synthesis, purification and in-line analytical units, and would need staff resource, i.e., a highly trained chemist, to keep it running. The consensus was such a machine is not too far away as advances in the last decade have made its construction much more tractable.

## Conclusions

Techniques for the computational analysis of individual reactions and for the design of synthetic routes are powerful and accessible. Even though there are many challenges remaining in both areas, the methods have developed sufficiently to be relevant now for the automated, efficient and sustainable manufacture of molecules old and new.

